# Toll-Like Receptor Expression and Responsiveness of Distinct Murine Splenic and Mucosal B-Cell Subsets

**DOI:** 10.1371/journal.pone.0000863

**Published:** 2007-09-12

**Authors:** Murali Gururajan, Joshy Jacob, Bali Pulendran

**Affiliations:** 1 Vaccine Research Center, Emory University, Atlanta, Georgia, United States of America; 2 Departments of Pathology, Emory University, Atlanta, Georgia, United States of America; 3 Microbiology and Immunology, Emory University, Atlanta, Georgia, United States of America; New York University School of Medicine, United States of America

## Abstract

**Background:**

Toll-like receptors (TLRs) are pattern recognition receptors that recognize pathogen associated molecular patterns and trigger innate immunity leading to initiation of adaptive immunity. TLR-mediated activation of dendritic cells (DCs) is known to be a critical event in the initiation of cellular and humoral immune responses. Recent work however suggests that B cells also express TLRs, and that they can be activated via TLR ligands. However, whether such B cell activation occurs only on memory B cells, or whether it can also occur on truly naïve B cells remains controversial. Furthermore, the expression and functional relevance of TLRs on distinct subsets of B cells, which are known to play differential roles in humoral responses is not known.

**Methodology/Principal Findings:**

In this study, we investigated the expression pattern of different TLRs in distinct subsets of murine B cells (naïve, memory, follicular, marginal zone, B-1 and peyer's patch). In contrast to the reported restricted expression pattern of TLRs in human peripheral blood naïve B cells, murine splenic naïve B cells express a variety of TLRs with the exception of TLR5 and 8. Consistent with this relatively broad expression pattern, murine naive B cells proliferate and secrete antibody to a variety of TLR agonists in vitro, in the absence of B-cell receptor cross-linking. In addition, we observed subtle differences in the antibody secretion pattern of follicular, marginal zone, B-1 and peyer's patch B-cell subsets.

**Conclusions/Significance:**

Thus various B cell subsets, including truly naïve B cells, express multiple TLRs, and signaling via such TLRs results in their robust proliferation and antibody secretion, even in the absence of dendritic cell activation, or T-cell help.

## Introduction

TLR-mediated recognition of microbial stimuli often leads to activation of innate immune cells including dendritic cells (DCs) [1], [2], [3]. TLR signaling promotes activation and maturation of DCs which instruct and support T-cell activation, leading to cell-mediated adaptive immune response [4]. Cognate interaction between activated antigen-specific T cells and naïve B cells promotes B-cell clonal expansion and differentiation leading to a humoral immune response. Differential expression pattern of TLRs have been reported in human and murine DC subsets [5], [6]. In humans, myeloid DCs express TLR2 and 4, whereas plasmacytoid DCs express TLR7 and 9 [7].

Recent studies suggests that in addition to TLR signaling in DCs, direct TLR mediated activation of B cells is also required for eliciting humoral immune response [8]. Thus chimeric mice in which only B cells are deficient in TLR signaling, fail to mount antibody responses to protein antigens given with adjuvants [8]. Consistent with this observation, previous work suggests that murine B cells can be stimulated in vitro by TLR4 and TLR9 ligands to proliferate and secrete antibody [9], [10]. ln contrast to murine B cells, naïve human B cells do not express TLR4 or TLR9 and hence do not respond directly to LPS or CpG [11]. However, it was recently reported that human memory B cells in the blood do express TLR9 and respond to CpG DNA [12], and consistent with this, cross-linking of BCR results in upregulation of TLR9 expression, and responsiveness to TLR9 ligands [12]. In contrast to this, recent studies revealed no differences in TLR expression in naïve versus germinal center versus memory B cells in human tonsils [13], [14].

Therefore, TLR activation of B cells likely plays a critical role in the regulation of humoral immunity and memory, and understanding the precise roles played by different TLRs in this regard is a critical first step in exploiting this in the design of vaccines that induce rapid and persistent neutralizing antibody. However, this is complicated by the fact that there are several subsets of B cells that play distinct roles during immune responses. For example, follicular B cells are shown to be important for T-dependent immune responses whereas marginal zone B cells are important for T-independent immune responses [15]. Marginal zone B cells are shown to be in a pre-activated state and respond rapidly to LPS and secrete antibody in vitro [16]. Peyer's patch B cells in the intestine play critical role in mucosal immune response by secreting IgA that binds to pathogens and prevents enteric infections [17]. B-1 B cells, a subset of B cells in the peritoneal cavity is the source of natural IgM present in the serum and plays an important role in immunity against blood borne pathogens [18]. However, the expression patterns of TLRs on distinct B-cell subsets, and their responsiveness to various TLR ligands is poorly understood.

To understand the importance of TLR signaling in B cells and to clarify the differences reported to exist between mouse and human B cells, we determined the expression pattern of TLRs in distinct murine B cell subsets and their response to TLR agonists in vitro in the absence of antigenic stimulation (BCR cross-linking). Our data suggest that mouse naïve follicular B cells express and undergo polyclonal expansion and differentiation to almost all known TLRs except TLR5 and 8. We demonstrate that different B-cell subsets including follicular, marginal zone, B-1 and peyers patch B cells proliferate and secrete polyclonal antibodies to a variety of TLR agonists in vitro.

## Materials and Methods

### Mice

C57BL/6 mice were purchased from Jackson Laboratory (Maine). ROSAYFP [19] and germinal center-cre (GCC) [19] mice were genotyped via PCR of genomic DNA derived from tail-tip biopsies. Primers for ROSAYFP PCR and Cre-PCR have been described [19] . All GCC mice were maintained by mating with C57BL/6 and housed under specific pathogen-free conditions at the Emory Vaccine Center. All animal studies were approved by the Institutional Animal Care and Use Committee of Emory University.

### Flow cytometry

Splenic B cells were bead purified using CD19 microbeads (Miltenyi Biotech). CD19 enriched B cells were stained for Cychrome anti-CD45R/B220 (RA3-6B2), PE anti-CD23 and FITC anti-CD21 (BD Pharmingen, San Jose, CA). B-cell subsets were FACS sorted using a MoFlo cell sorter (DakoCytomation, Fort Collins, CO).

### Real-time PCR

Total RNA was isolated from FACS sorted B cell subsets with the RNA miniprep (Invitrogen, Carlsbad, CA). RNA was quantified by OD_260_ using a DU® 530 Life Science UV Spectrophotometer (Beckman Coulter Inc, CA) and 2 µg of total RNA was subsequently used to make cDNA using the Superscript II reverse transcriptase (Invitrogen Corp., Carlsbad, CA) according to the manufacturer's protocol. RT-PCR was performed using Biorad apparatus (Biorad Laboratories, Hercules, CA). Primers for murine TLRs were used as reported previously [5]. The β-actin specific primers were used for loading control (IDT technologies, Corallevielle, IA).

### Proliferation Assay

FACS sorted B-cell subsets were incubated at 2×10^5^ cells/well in triplicate in 96-well flat-bottom plates in medium consisting of RBMI-1640 supplemented with 10 mM glutamine, 10 mM HEPES, 0.5 mg/ml gentamicin, and 5×10^−5^ 2-ME (Complete IF-12 media). Stimuli added included (Fab')_2_ goat anti-IgM (µ-chain specific; ICN Pharmaceuticals, Cappel, ICN Pharmaceuticals, Aurora, OH)) LPS (Invivogen), CpG ODN 1826 (Coley Pharmaceuticals, Wellesley, MA), Pam3Cys (EMC microcollections, Germany), MALP-2 (Invivogen, San Diego, CA), and 3M-003 (kind gift from Dr. Sefik Alkan, 3M pharmaceuticals, St. Paul, MN). After 66 h, all cultures were pulsed with 1 µCi of [^3^H]thymidine and harvested 6 h later onto filter mats using a cell harvester (Packard, Meriden, CT). The levels of radionucleotide incorporation were measured with a Matrix 96 ß-radiation counter (Packard, Downers Grove, IL). Results were presented as arithmetic mean of triplicate cultures ±SE.

### ELISA

FACS sorted B-cell subsets were incubated at 2×10^5^ cells/well in triplicate cultures in 96-well flat-bottom plates for 5 days. Day 5 supernatants were analyzed for antibody isotypes using ELISA kit (BD Biosciences, San Jose, CA).

### Immunization

C57BL/6 mice were immunized with sheep RBC (SRBC) 10% v/v intraperitoneally. On day 12, mice were sacrificed and B220^+^ PNA^−^ IgG^+^ splenic memory B cells FACS sorted using a MoFlo cell sorter (DakoCytomation, Fort Collins, CO) as described previously [20].

## Results and Discussion

### Expression profiles of TLRs in different B-cell subsets

**Figure 1:**
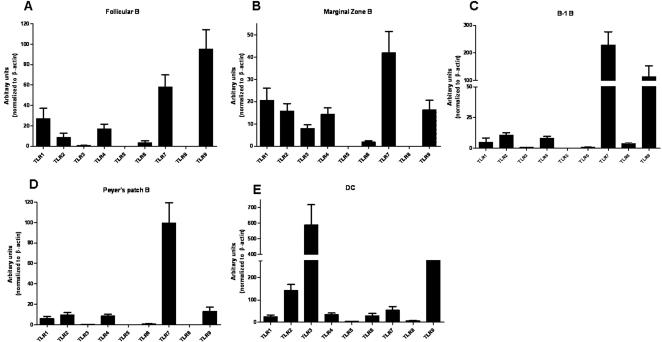
TLR expression profile of murine B-cell subsets. Real-time PCR profile of TLR expression in FACS sorted follicular B-cells (CD19^+^ B220^+^CD23^+^CD21^−^, panel A), marginal Zone B (CD19^+^ B220^+^CD23^−^CD21^+^, panel B), peritoneal B-1 (CD19^+^B220^+^CD23^−^, panel C) and peyer's patch B cells (CD19^+^B220^+^, panel D) as indicated with β-actin as loading control. CD11c+ dendritic cells were used as a positive control (panel E). Values represent the ratio of the TLR to β-actin.

We determined the expression pattern of TLRs1-9 in the following murine B-cell subsets i) splenic follicular ii) splenic marginal zone, iii) peritoneal B-1 and iv) mucosal peyer's patch B cells. In initial experiments, splenic follicular, marginal zone, peritoneal B-1 and peyer's patch B-cells were screened for expression of 9 known TLRs. Total B cells were isolated from murine spleen using CD19 microbeads and stained with antibodies to B220, CD23 and CD21 to distinguish different subsets. Splenic follicular (B220^+^CD23^+^CD21^−^) and marginal zone (B220^+^CD23^−^CD21^+^) B cells were sorted by cell sorting. The expression of different TLRs was assessed by real-time PCR with β-actin as loading control. All B-cell subsets expressed TLRs 1, 2, 3, 4, 6, 7 and 9 to varying degrees but not TLRs 5 and 8 (Figures 1A–D). As a positive control, CD11c^+ ^murine splenic DCs were used. CD11c^+^ DCs express all known TLRs (Figure 1E). B-1 B cells (B220^+^CD19^+^CD23^−^), a subset of B cells important for T-independent immune response present in the peritoneal cavity expressed a similar TLR pattern like splenic B cells except TLRs 1/2 and 6 (Figures 1C).

### Follicular, marginal zone, B-1 and peyer's patch B cells proliferate and secrete polyclonal antibodies to various TLR agonists

**Figure 2 pone-0000863-g002:**
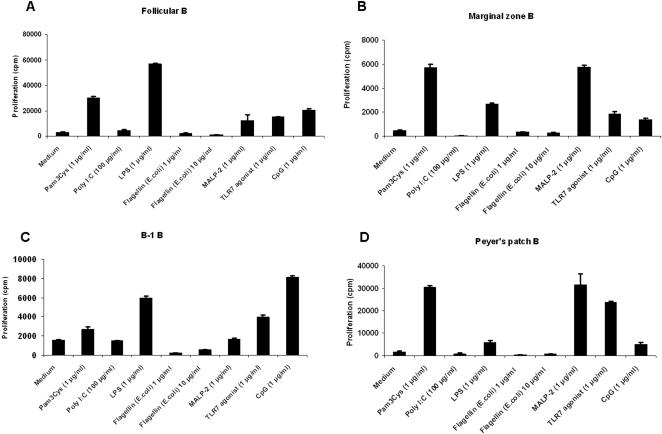
TLR-induced proliferation of murine B-cell subsets. A, FACS sorted follicular B-cells (CD19^+^ B220^+^CD23^+^CD21^−^) were cultured in vitro with various TLR ligands as indicated for 3 days and proliferation measured as described in Materials and Methods. B-D, FACS sorted marginal zone, B-1, and peyer's patch B-cells (CD19^+^ B220^+^CD23^−^CD21^+^ for marginal zone B, CD19^+^B220^+^CD23^−^ for peritoneal B-1 B and CD19^+^B220^+^ for peyer's patch B cells) were cultured in vitro with various TLR ligands as indicated for 3 days and proliferation measured by thymidine incorporation as described in Materials and Methods.

**Figure 3:**
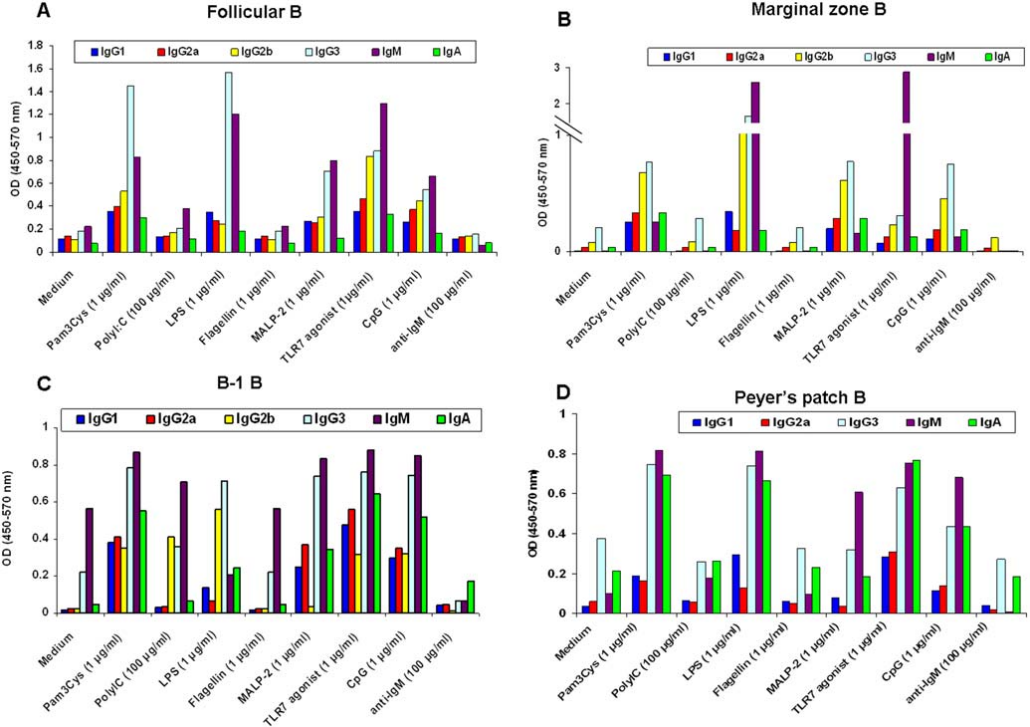
TLR-induced B-cell differentiation and immunoglobulin secretion of murine B-cell subsets. A, FACS sorted follicular B-cells (CD19^+^ B220^+^CD23^+^CD21^−^) were cultured in vitro with various TLR ligands as indicated for 5 days and antibody profile of culture supernatants measured by ELISA as described in Materials and Methods. B-D, FACS sorted marginal zone, B-1 and peyer's patch B-cells (CD19^+^ B220^+^CD23^−^CD21^+^ for marginal zone B, CD19^+^B220^+^CD23^−^for peritoneal B-1 B and CD19^+^B220^+^ for peyer's patch B cells) were cultured in vitro with various TLR ligands as indicated for 5 days and antibody secretion measured by ELISA as described in Materials and Methods.

First, we studied the functional relevance of TLR expression by various B-cell subsets. We found that murine B-cell subsets proliferate in response to 1 µg/ml of Pam3Cys (TLR2 agonist), LPS (TLR4 agonist), TLR2/6 (MALP-2), 3M-003 (TLR7/8 agonist) and CpG (TLR9 agonist) (Figures 2A-D) with varying degrees. Follicular B cells proliferate robustly in response to most of the TLR agonists except for TLR3 and TLR6, where the response is weak or absent and for TLR5 where the response is absent. Interestingly, marginal zone B cells proliferate robustly in response to TLR2 (Pam3Cys) and TLR6 (MALP-2) agonists but weakly to TLR4, 7 and 9 agonists. B-1 B cells respond weakly to most of the TLR agonists except TLRs 4, 7 and 9. Robust proliferation of peyer's patch B cells was observed in response to TLR2, 6 and 7 ligation but not other TLRs. Alternately, CFSE dilution of follicular B-cells in response to various TLR agonists was measured and B-cells appear to undergo multiple cell divisions in vitro (data not shown). The dose we used to stimulate B cells is optimal since dendritic cells stimulated with 1 µg/ml of TLR ligands secrete cytokines like IL-6, IL-12p40 and IL-10 depending on the stimulus [21], [22]. Follicular and marginal zone B cells not only clonally expand in response to TLR ligation but also secrete antibodies of all isotypes except IgA which was abundantly secreted by B-1 and peyer's patch B cells (Figures 3A-D). Consistent with their pre-activated phenotype, B-1 B cells have higher background IgM response and Peyer's patch B cells have higher IgA response in unstimulated conditions (Figure 3C & D) [18], [23]. The isotype profile appears to be different depending on the subset studied and the ligand used. Thus, our studies demonstrate that not only do different B-cell subsets express TLRs, but that they can also be stimulated directly by TLR ligands.

### Both naïve and memory murine B cells respond to TLR agonists in vitro in the absence of BCR cross-linking

**Figure 4:**
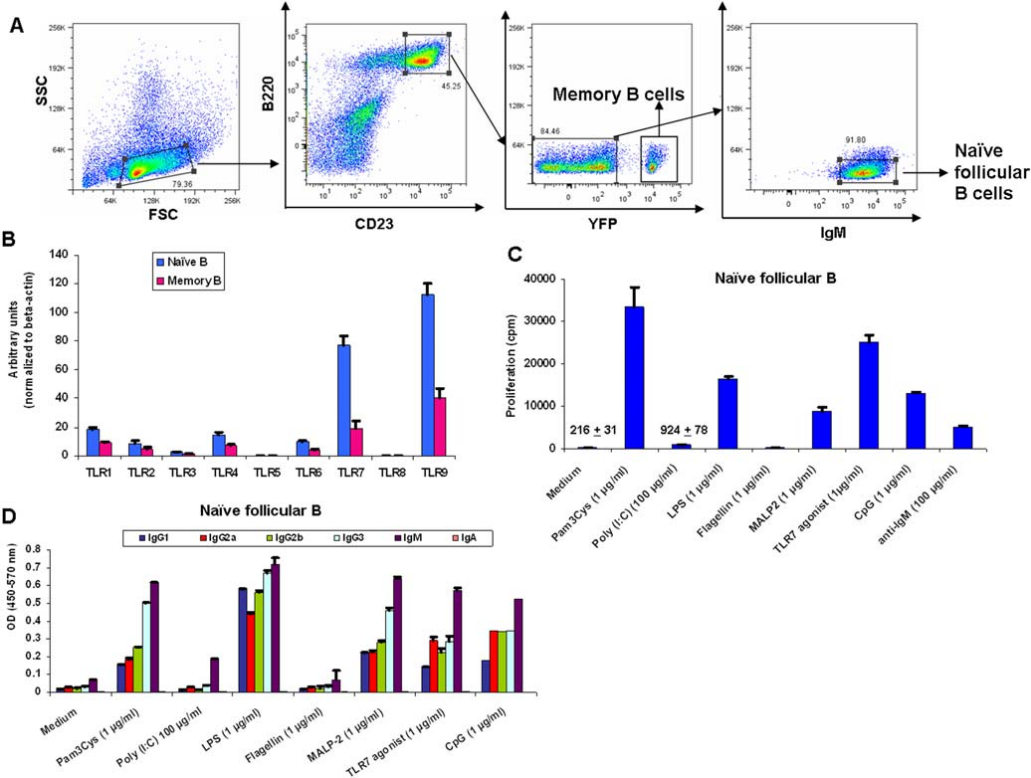
TLR induced proliferation and immunoglobulin secretion of naïve follicular B cells. A, Splenocytes taken from naive GCCxRosaYFP mice were stained with anti-B220-allophycocyanin and anti-CD23-phycoerythrin to detect follicular B cells. B220^+^ CD23^+^ cells were further gated for YFP and IgM expression. Numbers shown in each plot indicate frequency of gated cells within total lymphocytes. FACS profile of Naïve (B220^+^ CD23^+^ eYFP^−^ IgM^+^) B cells pre-sort and post-sort. The purity of post-sort was ∼99%. B, FACS sorted naïve B cells as described in panel A and memory B cells (B220^+^ CD23^+^ IgG^+^) from 10% v/v SRBC immunized mice (day 12) were probed for TLR expression by Real-time PCR, C, FACS sorted naïve B cells were cultured in vitro in the presence of various TLR ligands for 3 days and proliferation measured by thymidine incorporation as described in Materials and Methods. D, FACS sorted naïve B cells were cultured in vitro in the presence of TLR ligands for 5 days, culture supernatants were harvested and ELISA was performed as described in Materials and Methods. Experiments were performed three times with similar results and a representative profile is shown.

In humans, memory but not naïve B cells express and respond to TLR9 ligands in the absence of BCR triggering [12], [24]. However, in mice previous studies suggest that so-called naïve splenic B cells do respond directly to TLR stimulation [9], [10]. In our preliminary experiments, we sorted B220^+^CD23^+^CD21^− ^IgG^−^, putative naïve B cells and stimulated them in vitro with TLR ligands and observed proliferation and antibody secretion (data not shown). However it was formally possible that putative IgM^+^ memory B cells might have been present in the B220^+^CD23^+^CD21^− ^IgG^− ^fraction. To definitively address the issue of whether bona fide naïve B cells express TLRs and are responsive to TLR ligands, we utilized the recently described transgenic mouse system where memory B cells are marked based on YFP expression and can be tracked in vivo [19]. This transgenic system utilized the I-E*_α_* ^d^ promoter and gene expression from this promoter was restricted in B lineage cells that had entered the germinal center (GC) pathway of B cell differentiation. The transgenic mice were subsequently bred to the ROSAYFP cre-reporter strain, in which constitutive expression of YFP occurs upon cre-mediated recombination of the *ROSA*locus [19]. We FACS sorted naïve follicular (B220^+ ^CD23^+^ YFP^−^ IgM^+^) B cells from these transgenic mice thereby effectively excluding the contaminating memory B-cell population (Figure 4A). In parallel, we FACS sorted memory (B220^+^ CD23^+^ IgG^+^) B cells from sheep RBC (SRBC) immunized mice, and studied the expression and functional role for TLRs. Interestingly, the TLR expression profile of naive and memory B cells was almost identical (Figure 4B). Unlike human peripheral blood naïve B cells, murine splenic naïve B cells respond to TLR ligation (proliferation and antibody secretion) equally well compared to unsorted B cells, ruling out potential memory B cell contamination in the subsets studied (Figure 4C & D).

**Figure 5:**
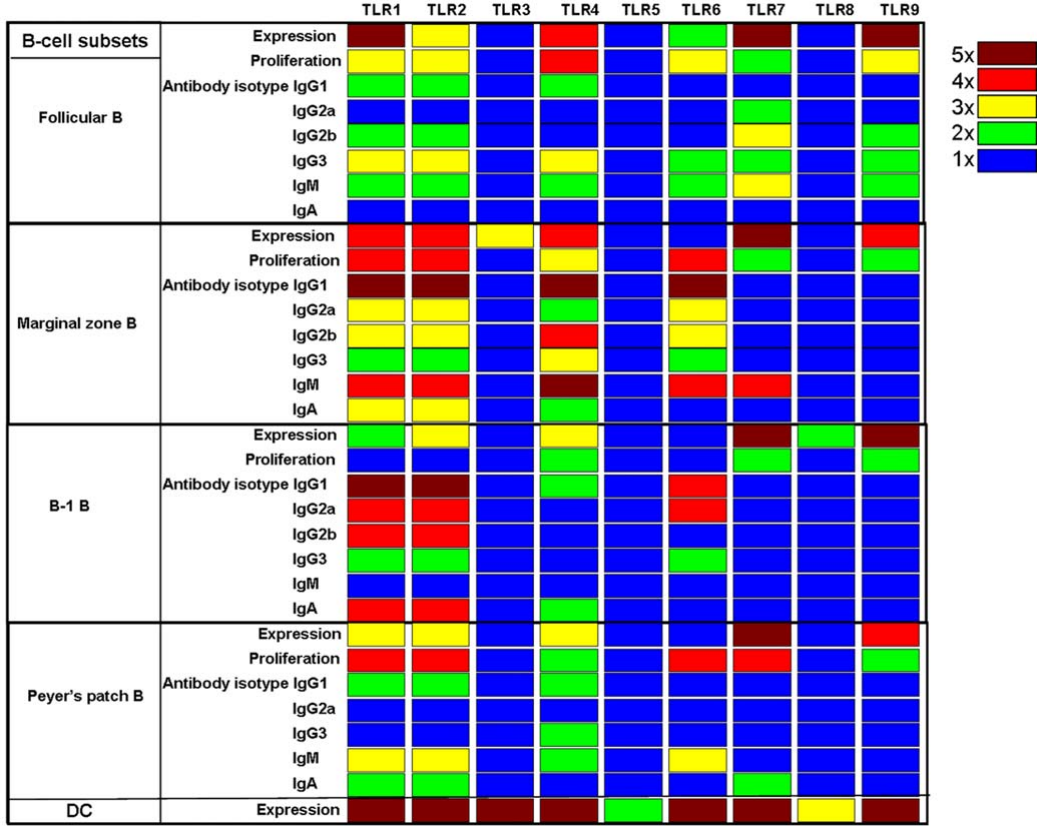
A heat map summary of TLR expression and responsiveness to different TLR ligands by distinct murine B cell subsets. The values for TLR expression profile, proliferation and antibody secretion in response to various TLR ligands by different B-cell subsets shown in Figures 1–3 were plotted on a log scale, and represented as a heat map. For TLR expression, blue represents an 1 fold increase relative to β-actin, green represents 2 fold increase, yellow represents 3 fold increase, red represents 4 fold increase and dark brown represents ≥5 fold increase. For proliferation, blue represents an 1 fold increase relative to unstimulated medium controls, green represents 2 fold increase, yellow represents 3 fold increase, red represents 4 fold increase and dark brown represents 5 fold increase. For antibody secretion, blue represents an 1 fold increase relative to unstimulated medium controls, green represents 2 fold increase, yellow represents 3 fold increase, red represents 4 fold increase and dark brown represents 5 fold increase.

In summary, we report here the expression pattern and function of TLRs in different murine B-cell subsets including follicular, marginal zone, B-1 and Peyer's patch. B-cell subsets express all known TLRs except 5 and 8. Consistent with their expression pattern, FACS sorted purified B-cell subsets respond to TLR ligands in vitro (Figure 5). Robust proliferation and antibody secretion of follicular B cells was observed in response to various TLR ligands, particularly ligands to TLR2/1, TLR2/6, TLRs 4, 7 and 9 (Figure 5). Marginal zone B cells have a similar pattern of TLR expression and response (Figure 5). However, B-1B cells do not respond strongly to TLR2 ligands. In contrast, Peyer's patch B cells respond robustly to TLR2, 6 and 7 ligation, but poorly to ligands for TLR9 and 4. Our results are consistent with a very recent report that B-cell subsets that participate in T-independent immune response express and respond to TLR agonists in vitro [25]. But in contrast to their observations, we show that follicular B cells that participate in T-dependent immune response do express and secrete antibodies of IgG3 and IgM isotypes in response to a variety of TLR agonists in vitro [25]. The observed differences between our study and the previous study could be due to different CpG motifs that were used (CpG 1826 versus CpG 1668). It is possible that the CpG ODN we used (CpG 1826) was more potent than the one used in the previous study (CpG 1668) [25], [26].

Furthermore, we addressed the issue of TLR expression and function in naïve versus memory B cells and found that mouse naïve follicular B cells express multiple TLRs and respond to a variety of TLR agonists in vitro. The extent to which TLR signaling in B cells modulate antibody responses to vaccines needs to be examined. Surprisingly, a recent report from Gavin et al demonstrated that certain commonly used adjuvants including CFA and Ribi do not require TLRs for eliciting antibody responses [27]. In contrast, it was shown that VSV induced IgM neutralizing antibody was partially dependent on TLR7 [28]. Furthermore, our recent study suggests that the yellow fever vaccine-17D, one of the most effective vaccines stimulates T cell responses by activating multiple TLRs [29], although to what extent TLR signaling influences humoral immune responses need to be determined.

In conclusion, the data presented here highlight the importance of TLR expression and function in different murine B-cell subsets which contribute to T-dependent and T-independent responses and signifies the potential fundamental differences that exist between human and mouse naïve B cells.
